# SPECT/CT Accurately Predicts Postoperative Lung Function in Patients with Limited Pulmonary Reserve Undergoing Resection for Lung Cancer

**DOI:** 10.3390/jcm13206111

**Published:** 2024-10-14

**Authors:** Isabelle Moneke, Christine von Nida, Oemer Senbaklavaci, Mirjam Elze, Philipp T. Meyer, Bernward Passlick, Christian Goetz, Laurin Titze

**Affiliations:** 1 Department of Thoracic Surgery, Faculty of Medicine, Medical Center—University of Freiburg, 79106 Freiburg im Breisgau, Germany; isabelle.moneke@uniklinik-freiburg.de (I.M.); oemer.senbaklavaci@usz.ch (O.S.); mirjam.elze@uniklinik-freiburg.de (M.E.); bernward.passlick@uniklinik-freiburg.de (B.P.); 2 Department of Nuclear Medicine, Faculty of Medicine, Medical Center—University of Freiburg, 79106 Freiburg im Breisgau, Germany; christine.von.nida@web.de (C.v.N.); philipp.meyer@uniklinik-freiburg.de (P.T.M.); christian.goetz@uniklinik-freiburg.de (C.G.)

**Keywords:** SPECT CT, lung cancer, planar perfusion scintigraphy, postoperative pulmonary function

## Abstract

**Background:** Preoperative prediction of postoperative pulmonary function after anatomical resection for lung cancer is essential to prevent long-term morbidity and mortality. Here, we compared the accuracy of hybrid single-photon emission computed tomography/computed tomography (SPECT/CT) with traditional anatomical and planar scintigraphy approaches in predicting postoperative pulmonary function in patients with impaired lung function. **Methods:** We analyzed the predicted postoperative pulmonary function in patients undergoing major anatomical lung resection, applying a segment counting approach, planar perfusion scintigraphy (PPS), and SPECT/CT-based lung function quantification. **Results:** In total, 120 patients were evaluated, of whom 82 were included in the study. Postoperative lung function tests were obtained in 21 of 82 patients. The preoperative SPECT/CT-based quantification yielded very accurate results compared to the actual postoperative FEV1 and DLCO values. The linear regression analysis showed that the SPECT/CT-based analysis predicted postoperative FEV_1_ (%) and D_LCO_ values more accurately than the segment counting approach or PPS. Accordingly, 58/82 patients would qualify for anatomical lung resection according to the SPECT-based quantification, 56/82 qualified according to the PPS (Mende), and only 47/82 qualified according to the segment counting method. Moreover, we noted that the SPECT-predicted FEV_1_ values were very close to the actual postoperative values in emphysema patients, and selected patients even showed improved lung function after surgery. **Conclusions**: Anatomically driven methods such as SPECT/CT yielded a very accurate prediction of the postoperative pulmonary function. Accordingly, applying SPECT/CT revealed more patients who would formally qualify for lung resection. We suggest SPECT/CT as the preferred method to evaluate eligibility for lung surgery in selected patients with impaired pulmonary reserve.

## 1. Introduction

In early-stage non-small cell lung cancer, surgical resection often provides the best chance for a cure [1]. To ensure an acceptable quality of life, preoperative evaluation and prediction of the remaining postoperative pulmonary function after anatomical resection is essential to reduce morbidity and mortality [2]. Patients suffering from pulmonary diseases such as chronic obstructive pulmonary disease (COPD) frequently present with impaired pulmonary function and have a high risk for severe intraoperative and postoperative complications [3,4]. Therefore, the thoracic surgeon needs to estimate the remaining lung function after anatomical resection (e.g., lobectomy) to decide which patients might be suitable for operative therapy. Preoperative lung function testing, evaluation of individual anatomy, and lung imaging procedures are commonly used to predict postoperative function in lung cancer patients.

The American College of Chest Physicians and the British Thoracic Society recommend using perfusion scintigraphy in patients with borderline lung function for both preoperative assessment and evaluation of expected residual pulmonary function after surgery [5]. The predicted postoperative function is usually defined as the remaining fraction of the preoperative pulmonary function. This definition may be extended to the predicted postoperative FEV_1_ (ppo-FEV_1_) or D_LCO_ (ppo-D_LCO_) [6]. When planning anatomical lung resection, according to the current guidelines [5,7], a postoperative value of 800 mL or ≥30% of the predicted reference value is usually desired for the FEV_1_ and ≥30% of the predicted reference value of the D_LCO_ [1,5].

Preoperative planar perfusion scintigraphy (PPS) quantification [8] and segment counting before lobectomy are well-established methods for predicting post-operative residual pulmonary function [7]. While they are easily implemented, they are also known to have a greater margin of uncertainty in projecting the lobar function [9], and they do not always give justice to lung lobe boundaries and, thus, to the patient’s anatomy [10,11]. Therefore, the Mende approximation of the planar evaluation is often used to avoid overlapping lung areas in planar perfusion scans [2,12] (Supplementary Figure S1) [2,12].

Combining the functional data provided by single photon emission computed tomography (SPECT) perfusion imaging with CT scan-based anatomical information could refine the preoperative quantification of lung function and improve the prediction of the residual pulmonary function after lung resection in selected patients (Supplementary Figure S2) [12,13].

This study aims to assess the accuracy of SPECT/CT regarding the preoperative prediction of post-surgery pulmonary function (ppo-FEV_1_ and ppo-D_LCO_) in patients with borderline lung function undergoing anatomical resection for lung cancer. The imaging results are compared to post-operative spirometry testing to evaluate the predictive value of the different approaches, focusing on patients with reduced pulmonary reserve and emphysema. We hypothesize that the use of anatomically driven SPECT/CT yields the most accurate predictions for postoperative lung function and might facilitate patient selection for surgery.

## 2. Methods

### 2.1. Design and Study Population

**Figure 1:**
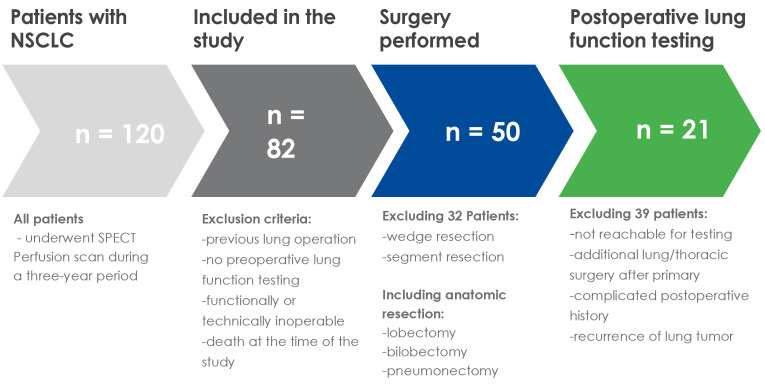
Diagram of patient selection.

We evaluated 120 consecutive patients with lung cancer and marginal lung function who were referred to our department for assessment of anatomical lung resection over three years. According to The European Society of Thoracic Surgery guidelines, operability was defined via the minimal expected post-operative values for FEV_1_ and D_LCO_ [5]. Patients with a history of previous lung resection and patients who were planned only for a wedge resection or a segmentectomy were excluded. In total, 82 patients technically qualified for major anatomical resection. After further assessment of the functional operability (e.g., cardiac evaluation and/or exercise testing and critical evaluation of alternative treatment options), surgery was performed on 50 patients. Pulmonary function testing was completed within one month before surgery. Postoperative spirometry testing after full recovery (3 months after surgery) was available for 21 patients. To compare the accuracy of the preoperative imaging techniques in predicting the postoperative lung function, we could only include patients with an uneventful postoperative course, e.g., patients who did not need to undergo another surgery or further treatment. The detailed patient selection process is shown in Figure 1.

### 2.2. Preoperative Imaging

Imaging (including low-dose CT, planar, and tomographic perfusion imaging) was performed using a double-head hybrid SPECT/CT system (Siemens Intevo 6 SPECT/CT, Siemens Healthcare GmbH, Erlangen, Germany) equipped with low energy high-resolution collimators. Technetium-99m-labeled macro aggregated albumin ([^99m^Tc] MAA; xx ± xx MBq) was injected shortly before SPECT/CT acquisition. The slow intravenous injection covered 2–3 breathing cycles, with the patient lying in the supine position and taking deep breaths. The perfusion SPECT consisted of a 360° acquisition in the supine position using a non-circular orbit with 64 projections (14 s per view; matrix: 128 × 128 pixels; energy window 140 keV ± 10%). During acquisition, all patients breathed shallowly; no respiratory gating was performed. Scatter correction (scatter window 110–130 keV) was applied before reconstruction by an iterative OSEM Flash 3D algorithm (8 subsets, 8 iterations, Siemens Syngo 6 Workstation, Siemens Healthcare GmbH, Erlangen, Germany). Subsequently, a low-dose CT was acquired in a mid-expiration position (5 mm slice thickness/pitch 0.85, tube voltage 110 kV, max current 25 mA, automatic exposure control system, matrix 512 × 512). The dataset was reconstructed with a slice thickness of 2.5 mm. Finally, 3 min planar perfusion images (anterior and posterior views) were acquired with a field of view of 55 × 40 cm (zoom = 1; matrix 256 × 256 pixels, energy window 140 keV ± 10%).

### 2.3. Spirometry

Spirometry assessment of pulmonary function was performed based on the standards of the European Respiratory Society (ERS). The measurements were repeated three times. The best of the three recordings was used for analysis. The forced expiratory volume in 1 s (FEV_1_), vital capacity (VC), residual volume (RV), and diffusing capacity for carbon monoxide (D_LCO_) were registered. The results were compared to the standard values for spirometry provided by the Global Lung Function Initiative (GLI). The standard values are based on the records of 97.759 healthy nonsmokers, aged 2.5–95 years, measured in 72 centers in 33 countries and collected by the European Respiratory Society and GLI in 2012 [14].

### 2.4. Quantification and Prediction of Postoperative Pulmonary Function

The predicted postoperative pulmonary function for each patient was calculated based on the preoperative functional measurements (FEV_1_ and D_LCO_) and the predicted functional lung fraction (%) to be resected (predicted by preoperative imaging):

(ppo-FEV_1_ (or ppo-DLCO) = FEV_1_ (or D_LCO_) × (100 − functional lung fraction to be resected))

For patients who underwent anatomical lung resection and post-operative pulmonary function testing, we compared the predicted and measured post-operative pulmonary function (ppo-FEV1 and ppo-DLCO) for each of the four tested methods:

(1) Assessment of pulmonary function after surgery by segment counting

A quick and common estimation of the fraction of resected lung tissue (%) is usually achieved by dividing the number of resected lung segments by 19 (total segments of both lungs) [15].

(2) Quantification based upon planar perfusion scintigraphy

Ventral and dorsal planar perfusion images were divided into three regions of interest (ROI) of equal height for each lung using the vendor’s application software (Siemens Healthcare GmbH, Erlangen, Germany). The upper and lower lung limits were set manually. The total counts collected from these images and for each ROI on the anterior and posterior views were processed using the Mende approach to assess the relative perfusion of each lobe [16]. The resected fraction (%) was estimated by dividing the counts within the lobes to be resected by the total counts in both lungs. (Supplementary Figure S2).

(3) Perfusion SPECT/CT-based lung function quantification

After transferring the co-acquired CT and perfusion SPECT/CT datasets to a commercially available quantification software (Hermia, Hybrid 3D Lung Lobe Quantification, Hermes Medical Solutions, Stockholm, Sweden) and after validation of the CT-based pulmonary lobes segmentation, the same volumes of interest (VOI) were transferred to the co-registered perfusion SPECT dataset. The relative perfusion (%) of each lung lobe was calculated by dividing the counts of each VOI by the summed counts of all lung lobes, as previously described [12].

### 2.5. Statistical Analysis

Statistical analysis was performed using SPSS Statistics 25 (IBM Corporation, Armonk, NY, USA). The mean values (standard deviation) of the functional fraction of each lung lobe were calculated and compared for each approach (segment counting, planar perfusion scintigraphy and SPECT/CT), and the respective differences were tested for significance employing the *t*-test for paired samples. The association of the predicted postoperative lung function values with the postoperatively measured values was assessed using linear regression analysis. The dependent variable is the postoperatively measured FEV_1_ (in % predicted) and D_LCO_ (in % predicted). The independent variable is the respective calculated postoperative predicted value (Segment counting, PPS and SPECT/CT). The variables are plotted using a scatter diagram, and a linear regression line is shown. Additionally, a line of identity is shown, which provides an additional reference when comparing the two datasets. On the line of identity, the postoperative predicted values equal the postoperatively measured values. A *p*-value ≤ 0.05 was considered statistically significant.

## 3. Results

In total, 82 patients (average age 68 ± 9 years, 63% male) were included in the study. Of these, 63% were diagnosed with COPD, 51% with cardiovascular diseases, and 18% with diabetes mellitus. The relevant patient characteristics are summarized in Table 1.

The UICC tumor stage for all included patients can be found in Supplementary Table S1. We estimated the postoperative lung function using the above-described established methods, e.g., a segment counting approach and methods based on computed tomography and scintigraphy. The preoperatively predicted values of the remaining pulmonary function after surgery are compiled in Table 2.

The results were quite similar between methods in most anatomical resections. While the predictions after the middle lobe resection showed the best concordance between all the methods for ppo-FEV_1_ and ppo-D_LCO_, slightly larger differences were observed between the segment counting method and the SPECT/CT in cases of pneumonectomy (left as well as right). Overall, the SPECT/CT perfusion scan yielded the highest percentage of predicted remaining postoperative lung function for most individual anatomical regions, as well as the calculated mean of all 82 patients (Table 2).

In total, 50/82 of our patient cohort underwent major anatomic lung resection; however, when we applied the formal criteria for functional operability, there were differences between the three methods. Applying the different approaches, 58, 56, and 47 patients qualified for anatomical lung resection by SPECT/CT, PPS, and segment counting, respectively. In total, 24 (48%) of the operated patients underwent neoadjuvant or adjuvant therapy (Supplementary Table S2).

Postoperative pulmonary function testing was available for 21/82 patients. Here, we analyzed the preoperative predicted values of each of the above-described methods and the actual values measured after anatomical resection. The linear regression analysis of the preoperative predicted values with the measurements obtained in the postoperative pulmonary function testing showed a significant correlation for each tested method (*p* < 0.01). Compared to the actual postoperative FEV_1_ and D_LCO_ values, the closest predicted post-operative pulmonary function values were yielded by SPECT/CT-based quantification (Table 3).

**Figure 2:**
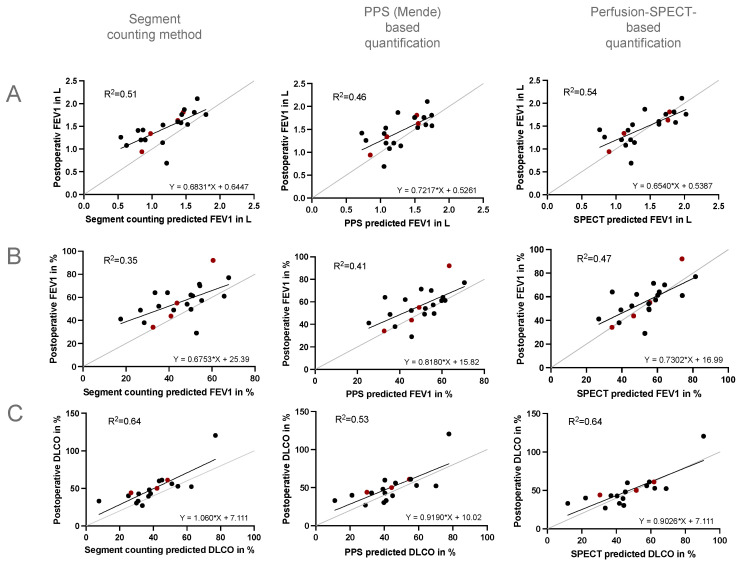
Simple linear regression analysis for predicted and postoperative FEV_1_ (L, %) (**A**,**B**) and D_LCO_ (%) (**C**). Grey: line of identity. Black: patients with postoperative lung function testing, except emphysema patients. Red: emphysema patients. Determination coefficient R^2^. FEV_1_: *n* = 21, D_LCO_*: n* = 18. PPS = planar perfusion scintigraphy.

The predicted preoperative values resulting from anatomy-based SPECT/CT analysis showed the strongest correlation with the postoperative FEV_1_ (L), FEV1 (%), and D_LCO_ of the patients, whereas the planar perfusion-based estimation was a little less accurate, and the segment counting method showed the weakest correlation. When comparing the determination coefficients (R^2^), the SPECT/CT-based predictions of the postoperative pulmonary function were the most accurate (Figure 2).

In the linear regression analysis of the postoperative and preoperative SPECT/CT-predicted FEV_1_ values, the subgroup of four patients with pulmonary emphysema appeared very close to the ‘line of identity’ suggesting the high accuracy of the SPECT/CT-predicted values in these patients (Figure 2). Moreover, most likely due to the lung volume reduction effect, lung function after resection may even improve in selected patients with pulmonary emphysema. Again, the SPECT/CT-predicted values were very accurate and came closest to postoperatively measured values (Table 4).

## 4. Discussion

In lung cancer patients who are technically eligible for resection, the thoracic surgeon needs to assess the functional operability to prevent significant morbidity and mortality. The gold standard in patients with borderline lung function has long been planar perfusion scan (PPS) [2,5]. However, data indicate that the SPECT/CT method might better predict the remaining postoperative lung function [8,13].

More than half of the 82 patients presented with secondary diagnoses, which may impact the functional operability. Almost all the patients were smokers, and not surprisingly, 63% of the patients were diagnosed with COPD, which mostly explains the marginal lung function. However, 51% suffered from additional cardiovascular diseases and 18% from diabetes mellitus. These data show the intricacy of making a statement regarding functional operability by solely looking at the aspect of lung function. Since we only had 21 patients with postoperative lung function, we could not perform additional statistical analyses to evaluate the impact of the respective comorbidity on postoperative pulmonary function.

The acquisition of postoperative lung function testing turned out to be one of the most challenging parts of the study. In total, 50/82 patients underwent surgery; however, due to the strict inclusion criteria, we were only able to obtain postoperative lung function tests in 21/82 patients. Following previous studies, we found that in our cohort, the SPECT/CT-predicted postoperative lung function seemed superior in accuracy for patients with marginal pulmonary function compared to the other established methods (e.g., the segment counting method and PPS). It is also the method that found the most patients eligible for surgery, suggesting that the other two methods tend to underestimate actual postoperative lung function. Accordingly, when we determined the ppo-FEV_1_ and ppo-D_LCO_ in all 82 patients, applying the SPECT/CT method resulted in fewer patients being considered functionally ineligible for surgery compared to the other two methods: an additional 11 (13.5%) patients would be assessed as functionally inoperable applying the segment counting method compared to the SPECT/CT-based calculation, and 2 (2.5%) more patients would be considered inoperable with the PPS-calculated predicted postoperative lung function. However, further evaluation regarding comorbidity is needed, as mentioned above, before deciding eligibility for surgery.

In the small subgroup of patients with pulmonary emphysema, linear regression analysis showed that the SPECT/CT-based perfusion quantification yielded the most accurate results as well. Here, lung function even improved postoperatively in three out of four patients, most likely due to the side effect of lung volume reduction. For patients with marginal lung function and pulmonary emphysema specifically, a 3D model, which is closer to the anatomical properties, might be valuable for the thoracic surgeon when evaluating functional operability.

## 5. Limitations of the Study

A major limiting factor is the small number of patients in this single-center study. Based on the criteria described above, we included 82 patients in the study, and after further clinical assessment, 50 patients underwent surgery. Due to the strict inclusion criteria, only 21/82 patients were suitable for postoperative lung function analysis, thus leaving a very small cohort for statistical analysis, which, therefore, needs to be seen as descriptive and does not allow for a definite conclusion. Moreover, a statement regarding a patient’s functional operability cannot be made based on the predicted postoperative pulmonary function alone. The general condition, secondary diagnoses, and the overall cardiovascular function of the patient need to be considered. Patients with marginal lung function have a high risk for postoperative pulmonary complications, which may impair lung function and impair the results of the accuracy of the predictions. We thus excluded patients with complicated postoperative courses to avoid selection bias and only compared the lung function tests from patients with an uneventful postoperative course to the preoperative predicted values. However, in clinical practice, these complications cannot be fully avoided and might lead to worse postoperative lung function than expected. Therefore, the individual risk of the patient should be taken into consideration.

Lastly, the study design does not allow for consideration of neo- and adjuvant therapy or the initial UICC stage of the lung tumor. Almost half of the operated patients underwent additional cancer therapy before or after surgery, which may affect both the operability and the postoperative pulmonary function. Due to the small number of patients in the respective groups, no further statistical analysis could be conducted here. However, additional studies are warranted.

## 6. Conclusions

SPECT/CT predicts postoperative pulmonary function more accurately than segment counting or PPS. In patients with limited pulmonary function, it should be the preferred imaging method to help the thoracic surgeon decide on the functional operability. This can be especially relevant in selected patients with emphysema, where less accurate predictive functional values yielded by traditional approaches may affect the eligibility for lung cancer surgery. Larger, multicenter studies are warranted to verify the benefit of SPECT/CT analysis when determining the operability in patients with limited pulmonary reserve.

## Figures and Tables

**Table 1:** Patient characteristics.

Variable	All Patients (82)	Patients with Postoperative Lung Function (*n* = 21)
Sex		
- Male - Female	49 (60%) 33 (40%)	10 (48%) 11 (52%)
Age	67 ± 9	66 ± 8
Comorbidity		
- Smoking - COPD - Emphysema - Cardiovascular disease - Diabetes mellitus	66 (80%) 52 (63%) 10 (12%) 51 (62%) 15 (18%)	18 (86%) 15 (71%) 4 (19%) 12 (57%) 3 (14%)
Resected area		
- RUL - RML - RLL - LUL - LLL - RUB - RPN - LPN	21 (26%) 3 (4%) 8 (10%) 14 (17%) 11 (13%) 2 (2%) 14 (17%) 9 (11%)	5 (24%) 0 (0%) 2 (10%) 4 (19%) 3 (14%) 1 (5%) 1 (5%) 5 (24%)

RUL = right upper lobe, ML = middle lobe, RLL = right lower lobe, LUL = left upper lobe, LLL = left lower lobe, RUB = right upper bilobectomy, RPN = right pneumonectomy, LPN = left pneumonectomy.

**Table 2:** Predicted remaining postoperative pulmonary function: FEV1 (pFEV_1_) and D_LCO_ (pD_LCO_).

	Scheduled Resection	*n* Patients	Segment Counting Method	Planar Perfusion Scintigraphy (Mende Method)	Perfusion-SPECT-Based Quantification
pFEV_1_	Resection of the upper lobe right	21	1.44 L (±0.4) 54% (±14%)	1.35 L (±0.4) 50% (±15%)	1.42 L (±0.4) 52% (±14%)
	Resection of the middle lobe	3	1.36 L (±0.7) 44% (18%)	1.40 L (±0.7) 45% (±19%)	1.35 L (±0.6) 44% (±16%)
	Resection of the lower lobe right	8	1.32 L (±0.5) 49% (±11%)	1.41 L (±0.4) 53% (±11%)	1.47 L (±0.5) 55% (±14%)
	Resection of the upper lobe left	14	1.21 L (±0.3) 46% (±8%)	1.29 L (±0.3) 50% (±10%)	1.34 L (±0.4) 52% (±12%)
	Resection of the lower lobe left	11	1.34 L (±0.4) 51% (±13%)	1.28 L (±0.4) 49% (±15%)	1.47 L (±0.5) 56% (±19%)
	Resection of two upper lobes right	2	1.32 L (±0.2) 48% (±6%)	1.24 L (±0.3) 45% (±0.8%)	1.30 L (±0.1) 48% (±7%)
	Pneumonectomy right	14	0.84 L (±0.34) 31% (±11%)	1.03 L (±0.5) 38% (±15%)	1.08 L (±0.6) 39% (±17%)
	Pneumonectomy left	9	0.94 L (±0.4) 33% (±9%)	1.31 L (±0.5) 46% (±12%)	1.34 L (±0.5) 48% (±13%)
	Remaining lung function	82	1.22 L (±0.4) 45% (±14%)	1.28 L (±0.4) 47% (±13%)	1.34 L (±0.4) 50% (±15%)
pD_LCO_	Resection of the upper lobe right	21	42% (±13%)	40% (±13%)	42% (±15%)
	Resection of the middle lobe	3	45% (±11%)	46% (±11%)	45% (±14%)
	Resection of the lower lobe right	8	39% (±10%)	42% (±10%)	43% (±11%)
	Resection of the upper lobe left	14	40% (±15%)	43% (±16%)	44% (±17%)
	Resection of the lower lobe left	11	43% (±14%)	40% (±14%)	46% (±17%)
	Resection of two upper lobes right	2	33% (±0.3%)	31% (±3%)	33% (±0.2%)
	Pneumonectomy right	14	23% (±7%)	28% (±13%)	29% (±14%)
	Pneumonectomy left	9	29% (±10%)	41% (±16%)	42% (±16%)
	Remaining lung function	82	36% (±13%)	39% (±14%)	41% (±15%)

pFEV_1_ and pD_LCO_ values are expressed in liters and percentage (%) of the expected age-, sex-, and body height-dependent values. The differences in the calculated predicted values are given in % of standard deviation (SD) for each method (brackets).

**Table 3:** Comparison of the predicted postoperative lung function to the actual postoperative remaining lung function. Calculation of the FEV_1_ and D_LCO_ for the respective methods. *n* = 21. Values are expressed in liters and percentages (%) of the expected age-, sex-, and body height-dependent value. The differences in the calculated predicted and the measured values are given in % of SD for each method (brackets).

	Predicted Postoperative Lung Function			Measured Remaining Lung Function
	Segment Counting Method	Planar Perfusion Scintigraphy (Mende Method)	Perfusion-SPECT-Based Quantification	
FEV_1_	1.1 L (±0.3) 45% (±13%)	1.3 L (±0.3 SD) 49% (±12%)	1.4 L (±0.4) 53% (±14%)	1.4 L (±0.3) 56 % (±15%)
D_LCO_	39% (±14.4%)	43% (±15%)	47% (±17%)	49% (±20%)

**Table 4:** Preoperative predicted and postoperative values of patients with pulmonary emphysema.

		Segment Counting Method	Planar Perfusion Scintigraphy (Mende Method)	Perfusion-SPECT-Based Quantification	Postoperative Values
1	FEV_1_ (L) FEV_1_ (%) D_LCO_ (%)	1.46 60 42	1.53 63 44	1.78 74 52	1.81 92 50
2	FEV_1_ (L) FEV_1_ (%) D_LCO_ (%)	1.38 44 49	1.55 49 55	1.76 56 62	1.63 55 61
3	FEV_1_ (L) FEV_1_ (%) D_LCO_ (%)	0.98 41 27	1.09 46 30	1.12 47 30	1.34 44 44
4	FEV_1_ (L) FEV_1_ (%) D_LCO_ (%)	0.85 33 38	0.85 33 (38)	0.89 34 (40)	0.94 34 ∅

Calculation of the predicted FEV_1_ and D_LCO_ for the respective methods and results of the actual postoperative lung function testing. *n* = 4.

## Data Availability

Data analyzed in this article will be provided upon reasonable request to the corresponding author.

## Supplementary Materials

The following supporting information can be downloaded at: https://www.mdpi.com/article/10.3390/jcm13206111/s1.
