# Supplementary material for: SPECT/CT Accurately Predicts Postoperative Lung Function in Patients with Limited Pulmonary Reserve Undergoing Resection for Lung Cancer
Source: J Clin Med. 2024 Oct 14;13(20):6111. doi: 10.3390/jcm13206111 (PMC11509096; doi:10.3390/jcm13206111)
Supplement: Supplementary file 1 [file jcm-13-06111-s001.zip › jcm-3195525-supplementary.pdf]

Supplementary Figure S1

Lung Lobe	Calculation
**RUL**	**Upper and middle ROI anterior, upper ROI posterior (right)**
**ML**	Lower ROI anterior (right)
**RLL**	Middle and lower ROI posterior (right)
**LUL**	Upper and middle ROI anterior, upper ROI posterior (left)
**LLL**	Lower ROI anterior, middle and lower ROI posterior (left)

Supplementary Figure S1*:* Mende approach based on the detected counts via planar perfusion scintigraphy. RUL= right upper lobe, ML = middle lobe, RLL= right lower lobe, LUL= left upper lobe, LLL= left lower lobe

Supplementary Figure S2

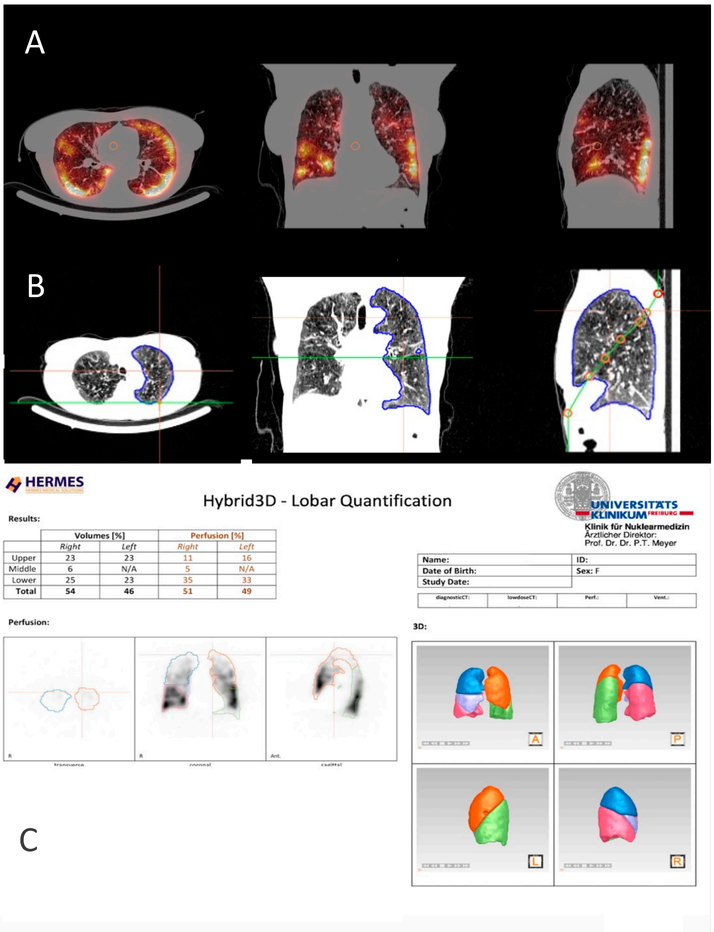

Supplementary Figure S2: Exemplary SPECT/CT with Hybrid 3D Lung Lobe Quantification (HERMES Medical Solutions, Stockholm). A: Fusion of CT scan images with SPECT data. B: Detection of the pulmonary lobe borders of the left lung in sagittal image orientation. C: Fraction of volume (%) and perfusion (%) for the respective lung lobes

Supplementary Table S1 – TNM Classification 8th edition of the 82 patients included in the study

Tumor	UICC- Stage	TNM Classification	N=82
	0	Tis N0 M0	
	IA1	T1a N0 M0	7 (9%)
	IA2	T1b N0 M0	7 (9%)
	IA3	T1c N0 M0	5 (6%)
	IB	T2a N0 M0	6 (7%)
	IIA	T2b N0 M0	6 (7%)
	IIB	T1a N1 M0	
		T1b N1 M0	2 (2%)
		T1c N1 M0	
		T2a N1 M0	1 (1%)
		T2b N1 M0	1 (1%)
		T3 N0 M0	5 (6%)
	IIIA	T1a N2 M0	2 (2%)
		T1b N2 M0	2 (2%)
		T1c N2 M0	1 (1%)
		T2a N2 M0	5 (6%)
		T2b N2 M0	2 (2%)
		T3 N1 M0	5 (6%)
		T4 N0 M0	7 (9%)
		T4 N1 M0	4 (5%)
	IIIB	T1a-c, T2a-b N3 M0	
		T3 N2 M0	4 (5%)
		T4 N2 M0	3 (4%)
	IIIC	T3 N3 M0	
		T4 N3 M0	1 (1%)
	IVA	TX NX M1a	2 (2%)
		TX NX M1b	2 (2%)
	IVB	TX NX M1c	
	N/A	N/A	2 (2%)

Table S1: There was no information regarding the initial TNM stage for two patients. UICC: Union Internationale Contre le Cancer, TNM: Classification 8th edition (Rami-Porta et al., 2015)

Supplementary Table S2 – Additional lung cancer therapy of the operated patients

**Additional Therapy**		N=50
Neoadjuvant	Chemotherapy	4 (8%)
	Chemoradiation	3 (6%)
Adjuvant	Chemotherapy	7 (14%)
	Radiotherapy	2 (4%)
	Chemoradiation	6 (12%)
	Targeted therapy	2 (4%)
No additional therapy		26 (52%)
